# Sir Geo. Ballingall's Lectures on Military Surgery

**Published:** 1833-04-01

**Authors:** 


**OUTLINES OF THE COURSE OF LECTURES ON MILITARY SURGERY, DELIVERED IN THE UNIVERSITY OF EDINBURGH. By Sir *George Ballingall*, MD. FRSE. Regius Professor of Military Surgery, &c. &c. 8vo. pp. 589.**

THIS is a very interesting volume; Sir George Ballingall is already favourably known to the profession by former writings, and the present work will not derogate from his literary or his professional reputation. At this late period of the quarter we cannot do more than offer a notice of some particular portions of the volume, and cannot profess to select all that is worth selecting.

Sir George observes that the arrangement which he has adopted in his Course of Military Surgery is arbitrary, but that he has found it satisfactory and convenient. The course consists of three divisions. The first embraces topics connected with the formation, discipline, and economy of armies, highly important to the health of soldiers. The second, comprising the great body of the course, embraces those surgical accidents and diseases peculiarly incident to military and naval men. The third division includes the diseases incident to troops on foreign stations, and those semblances of disease which are often more difficult to combat than the reality. As we have observed, we can only notice particular portions of the work, and its necessarily general elementary character also renders this the preferable plan. The first subject to which we shall allude is that of the diseases of camps and garrisons, and the proportion of sick in armies.

*Diseases of Camps and Garrisons.* It has been observed, says Sir George Ballingall, and perhaps justly, that in all large armies more men perish by inbred disease than by the sword of the enemy, and that more campaigns have been decided by sickness than by battle. Many of our readers may probably remember the sarcastic remarks of Voltaire on the prevalence of syphilis in armies. The diseases of soldiers have, for convenience sake, been divided into diseases of the camp and of the garrison. The former have also been characterized as the diseases of Summer and of Autumn, this being the season of the year for encampment. Here fever and dysentery have been in all ages the scourge of armies.

"Of the extent to which these two diseases prevailed in the Peninsular army, some conception may be formed from the following statement extracted from Sir James M'Grigor's account of the diseases of that army. There were admitted during the years 1812-13, and part of 1814, sixty-eight thousand eight hundred and ninety-four cases of fever, of which six thousand seven hundred and three died, equal to 9.7 per cent.; and during the same period there were admitted into the regimental hospitals seven thousand five hundred and twenty-six cases of dysentery, of which four thousand seven hundred and seventeen died, or 62.5 per cent." 63.

The diseases incident to soldiers in garrison or in quarters, which have also been characterized as the diseases of Winter and of the early part of Spring, are chiefly in this part of the world, inflammatory, as affections of the chest, rheumatism, ophthalmia; we must add, the venereal. In estimating the proportional sickness among the troops at different seasons of the year, Sir John Pringle has given the following result of his experience in Germany, Flanders and in this country, during the campaign of 1742, and the subsequent years.

"In the beginning of every campaign," says he, "we are to expect, for the first month at least, that the returns will be considerably higher than if the men had remained in quarters. The earliest encampment began on the 8th of April, and produced such a number of sick, that in a month's time the returns amounted to a twenty-seventh part of the whole. In the year 1745, the campaign was opened on the 25th of April; and in 1747, on the 23d of the same month, both in the *Low Countries*, but in the year 1746, the troops encamped on the 23d April in the north of *Scotland*, which, considering the latitude, may be reckoned the earliest campaign during the war. And from all these instances, there is reason to believe, that the first proportion mentioned will generally hold when the army takes the field in *Flanders* in the first or second week of

April. At the end of the campaign in *Germany*, the number in the hospital were to the men in health as three to thirteen. In 1747, when the troops left the field, the sick made about one-fifth part of the whole number, but if we consider by itself the detachment sent that year into *Zealand*, this proportion was just inverted; for the men in health were to the diseased only as one to four." We learn also from the same authority, that of the troops stationed, during 1747, in South Beveland and the Island of Walcheren, some of the corps were so sickly as not to have more than one hundred men fit for duty, which was less than the seventh part of a complete battalion ; " the Royals, in particular, at the end of the campaign, had but four men that never had been ill." 64.

This fatality in Walcheren has since been significantly commented on in the same pestiferous spot, where nearly four thousand men were lost. It must obviously, as Sir George Ballingall observes, be a difficult matter to calculate, before hand, on the mortality likely to ensue in any given service, so much must depend on accidental circumstances, such as the character of the season, the state of the provisions and lodging of the men, their morale, and so on. The most recent statement we possess of the actual mortality and contingencies incident to an European army, fighting on an European soil, has been given us by Sir James M'Grigor. Some error appears to have crept into the numbers. The statement is of the sick and wounded in the Peninsular army, from the 21st Dec. 1811, to the 24th June, 1816, embracing, of course, a period of very active operations.

" ' On reference to the returns of sick and wounded for the above period, it appears that three hundred and forty-six thousand one hundred and eight cases of disease or wounds were treated in the hospitals : of which were discharged cured two hundred and thirty-two thousand five hundred and fifty-three; four thousand five hundred and eighty-six were invalided; and eighteen thousand five hundred and thirteen died of their wounds or of disease ; including however every wounded man who had been received into hospital, or who had even been seen by a surgeon.' "

M. Vaidy, the author of an article on Hygiene Militaire, in the Dictionnaire des Sciences Medicales, states that in garrison in a healthy country, where provisions are abundant and the barracks well constructed, we may expect about five per cent. of sick in the infantry, and somewhat less in the cavalry and artillery. It is curious to observe a fact, which, however, has been often noticed, that the morale of the men makes the utmost difference in respect to health or sickness. Thus, M. Vaidy remarks that, after a successful campaign, he has known this proportional number much reduced. After the battle of Austerlitz, for instance, the French army, cantoned in Bavaria, had only a hundred sick in a division of eight thousand men.

" During a campaign, however, we ought not to calculate upon less than ten sick for every 100 fighting men, a number which is often fearfully increased if the army is very numerous and much concentrated, if it is encamped on wet ground, if it has experienced great privations, and finally, if it is discouraged by defeat or want of confidence in its chiefs. An army of 100,000 men may expect, according to Vaidy's calculation, to have 10,000 sick during a campaign, independent of any rencontre with the enemy ; of which number 5000 or 6000 may be medical and the rest surgical cases ; but after a battle, the proportion will be reversed, and under the most favourable circumstances, he calculates upon 10,000 or 12,000 wounded, in addition to the above. To this we have sometimes to add the number of wounded left in our hands by a vanquished enemy, to whom our cares are equally due. These calculations become of great importance to medical officers, particularly as they rise upwards in the gradation of ranks, and come eventually to be consulted as to the extent of the necessary establishments for the sick, or to regulate the proportion of medical staff and hospital stores to be allotted for any given service." 66.

Such we may expect in accompanying an army to the field. But we learn, both from ancient and modern wars, that sickness is not a necessary accompaniment, at least to a great extent, of warfare. Our army in the Peninsula was exposed to fruitful sources of disease, and sometimes suffered severely from it; yet, owing to the excellent arrangements of Sir James M'Grigor, and the medical staff under his orders, it remained in a most creditable state of efficiency. During the ten months from the siege of Burgos to the battle of Vittoria, inclusive, the total number of sick and wounded, which passed through the hospitals, was ninety-five thousand, three hundred and forty-eight. The army took the field preparatory to the battle, with a sick list under five thousand. For twenty successive days it marched towards the enemy; and in less than one month after it had defeated him, mustered within thirty men as strong as before the action, and without reinforcement from England, the ranks having been recruited by convalescents.

In the navy, the powers of prophylactic measures are most conspicuous. A long sea voyage was once considered a severe ordeal; but now a ship's company may be conducted round the world, with a smaller proportional loss of men than would happen, perhaps, under any other circumstances, or in any other given situation. Lord Nelson is said to have kept the crew of a vessel which he commanded in such perfect health, by his judicious regulations, that he did not lose a man by death in *three years*, and that on the West India station.

" The name of this distinguished officer, thus honourably connected with the health of his crew, reminds me that although it belongs to the medical officer to suggest measures for preserving the health of seamen and of soldiers, it belongs to the commanding-officer to give them due effect. The means of preserving the health and efficiency of troops has not yet sufficiently engaged the attention of the authorities in the British army, although the superiority of prophylactic over remedial measures has been distinctly pointed out by numerous writers, and must never be lost sight of. Sir Gilbert Blane remarks, that it could be made evident in an economical and political point of view, independent of moral considerations, that the health and lives of men might be preserved at a much less expense than what is necessary to repair the ravages of disease; and Sir John Pringle has observed, that 'although most of the causes of disease are hardly to be avoided in time of actual service, yet as these only dispose men to sickness, and do not necessarily bring it on, it is incumbent on those who have the power to make such provision as shall enable the soldier to withstand most of the hardships of a military life;' and, he adds with much truth, 'that the preservatives from disease are not to depend on *medicines*, nor on any thing which a soldier has it in his power to neglect.' " 68.

### Hospitals.

We are tempted to extract a few particulars from a very interesting account, which Sir G. Ballingall gives, of the origin and objects of military hospitals. We need hardly say that the ancients, behind us in most of the refinements and comforts of civilization, were not acquainted with the value of a regulated system of military surgery, or with the establishment of military hospitals. Honours were granted to the surviving wounded; they were laurelled when living, or deified when dead, but the sober task of curing their wounds, and ministering to their maladies, appears to have been forgotten or despised in the glare of the triumph or solemnities of the apotheosis.

" The first traces of field hospitals, or, as they are sometimes called, flying hospitals, occur perhaps in the east. At any rate, the Emperors Mauricius and Leo VI. had along with their armies certain followers termed *deputati*, (Δαιποταται,) who were distributed among the cavalry, and were obliged to carry off those wounded in battle. On this account they had on the left side of the saddle two stirrups, in order that they might more easily take up the wounded behind them; and for every person thus saved they obtained a certain reward. They were obliged also to carry with them a bottle containing water, for the purpose of reviving those who might have fainted through loss of blood." 73.

In point of fact, the chief object of attention appears to have been the spiritual, rather than the bodily wants of the wounded. By an order of the Council of Ratisbon, in 742, every commander was obliged to have with him two bishops, with priests and chaplains; and every colonel was saddled with a confessor, but no mention is made of field hospitals or of army surgeons. These barbarous marauders, for after all they were little better, that spilt oceans of human blood for an ecclesiastic dogma—that committed more atrocities than ever savages were accused of, in their religious crusades, these barbarians were thus intent on providing for the welfare of their souls, but seemed to think little of the healing of bodies.

" Harte, in his Life of Gustavus Adolphus, seems to believe that this prince first appointed four surgeons to each regiment, which he reduced from the number of 2000 or 3000, first to 1200, and afterwards to 1008; and Harte is of opinion that the imperial troops, at that time, had no surgeons, because Tilly himself, after the battle at Leipsic, was obliged to have his wounds dressed by a surgeon established at Halle. However this may be, it is certain, that the field hospital establishments of the imperial army, till the beginning of the 18th century, were on a very bad footing. Even in the year 1718, they had no field surgeons; but at this period the company surgeons were dismissed, and a regimental surgeon, with six assistants, was appointed to each regiment; and besides the field medicine chest, surgical instruments were provided at the emperor's expense." 74.

Field hospitals would appear to have been established in Germany, prior to the middle of the 16th century. According to Fronsperger's statement, it was necessary that there should be, along with the Commander-in-Chief, a field surgeon-in-chief, a doctor, who had the inspection of the field surgeons, the barbers, and their servants, whose duty was to drag the wounded from the heaps of slain, and to convey them to their masters. He was obliged to keep by him instruments and medicines, and at each muster to examine the instruments and apparatus of the field surgeons; he decided also in disputed cases, how much soldiers, whose wounds had been cured, ought to pay to the field-surgeon; and during marches he was bound to remain with the commander-in-chief. Fronsperger says also, that there ought to be with the artillery a general field-surgeon, and with each company a particular field-surgeon, not, however a paltry beard-scraper, *(bartscherer)* but a regularly instructed, experienced, and well practised man. Field hospitals were first established in France under Henry IV. at the siege of Amiens, in 1597, and so delighted were the soldiers that they called this the *velvet campaign*. However, even so late as the time of Dr. Brocklesby the accommodation provided for sick soldiers was most wretched. He observes that, to the important subject of military hospitals, " neither Montecuculli, Folard, Feuquieres, the great Conde, Marshal Saxe, General Bland, nor any other writer with whom he was acquainted, had paid much attention; for officers in this respect conceived that they had little more to do than to consign the sick to the best of those accommodations which chance, necessity, or a base parsimony, had provided for them." Sir G. Ballingall remarks that the present arrangements are most excellent, and proceeds to enumerate them. We wish we could accompany him in his visit to the military hospitals, but, unfortunately, we must here halt, and reserve any further operations for another season.

